# UNMET CARE NEEDS DURING THE COVID-19 PANDEMIC

**DOI:** 10.1093/geroni/igad104.2055

**Published:** 2023-12-21

**Authors:** Angela Curl, Amy Roberts

**Affiliations:** Miami University, Oxford, Ohio, United States; Miami University, Oxford, Ohio, United States

## Abstract

**Background:**

Few studies have examined the characteristics of individuals who experienced unmet care needs during the COVID-19 pandemic (a time when more needs may have gone unmet).

**Methods:**

Using 2020 data from the Health and Retirement Study, this study selected a sample of respondents over the age of 50 who reported difficulty with completing activities of daily living (ADL; eating, bathing, dressing, toileting, toileting; N=685). Unmet ADL need (outcome) was defined as having difficulty performing one or more ADLs but not receiving help performing that ADL. A logistic regression analyzed predictors of unmet ADL need, using the predictors of cognitive ability (range 0-27; Crimmins et al., 2011), self-rated eyesight, self-rated hearing, education, age, gender, race, marital status, and having a child living within 10 miles. Findings: Females were less likely to report an unmet ADL need (OR=0.66, p=.03), as were married/partnered respondents (OR=.52, p<.01). In contrast, respondents with higher cognitive ability were more likely to report unmet ADL needs (OR=1.04, p=.02). The remaining predictors were not statistically significant.

**Discussion:**

Having difficulty with basic ADLs and lacking assistance through formal or informal caregiving can increase the risk for a serious adverse event or nursing home placement. While those with higher cognitive ability could make use of creative workarounds (e.g., assistive devices); they may also have been perceived as having less need of assistance from others. By identifying groups who are more likely to have unmet ADL needs, outreach, assessment, and intervention outreach efforts can be targeted to those at greatest risk.

